# Paediatric cardiopulmonary resuscitation training program in Latin-America: the RIBEPCI experience

**DOI:** 10.1186/s12909-017-1005-1

**Published:** 2017-09-12

**Authors:** Jesús López-Herce, Martha M. Matamoros, Luis Moya, Enma Almonte, Diana Coronel, Javier Urbano, Ángel Carrillo, Red de Estudio Iberoamericano de estudio de la parada cardiorrespiratoria en la infancia (RIBEPCI), Jimena del Castillo, Santiago Mencía, Ramón Moral, Flora Ordoñez, Carlos Sánchez, Lina Lagos, María Johnson, Ovidio Mendoza, Sandra Rodriguez

**Affiliations:** 1 Paediatric Intensive Care Unit, Gregorio Marañón General University Hospital, Paediatrics Department, Faculty of Medicine, Complutense University, Madrid, Dr Castelo 47, 28009 Madrid, Spain; 2 Health Research Institute of the Gregorio Marañón Hospital, Madrid, Spain; 3 Mother-Child and Developmental Health Network (Red SAMID), Subdirección General de Evaluación y Fomento de la Investigación y el Fondo Europeo de Desarrollo Regional (FEDER) referencia Instituto de Salud Carlos III RD12/0026/0001, Madrid, Spain; 4 Hospital Escuela, Tegucigalpa, Honduras; 5 Hospital General San Juan de Dios, Ciudad de Guatemala, Guatemala; 6 Hospital General Plaza de la Salud, Santo Domingo, Dominican Republic; 7 Centro Nacional para la Salud de la Infancia y la Adolescencia, México, Distrito Federal Mexico; 8 Science and Technology Program for Development (CYTED), Madrid, Spain; 9 Spanish Agency for International Cooperation (AECI), Madrid, Spain; 10 Hospital General Universitario Gregorio Marañón, Madrid, Spain; 11 Hospital del Niño, Ciudad de Panamá, Panamá

**Keywords:** Resuscitation education, Pediatric resuscitation, Resuscitation training, Resuscitation courses, Pediatric life support courses

## Abstract

**Background:**

To describe the design and to present the results of a paediatric and neonatal cardiopulmonary resuscitation (CPR) training program adapted to Latin-America.

**Methods:**

A paediatric CPR coordinated training project was set up in several Latin-American countries with the instructional and scientific support of the Spanish Group for Paediatric and Neonatal CPR. The program was divided into four phases: CPR training and preparation of instructors; training for instructors; supervised teaching; and independent teaching. Instructors from each country participated in the development of the next group in the following country. Paediatric Basic Life Support (BLS), Paediatric Intermediate (ILS) and Paediatric Advanced (ALS) courses were organized in each country adapted to local characteristics.

**Results:**

Five Paediatric Resuscitation groups were created sequentially in Honduras (2), Guatemala, Dominican Republican and Mexico. During 5 years, 6 instructors courses (94 students), 64 Paediatric BLS Courses (1409 students), 29 Paediatrics ILS courses (626 students) and 89 Paediatric ALS courses (1804 students) were given. At the end of the program all five groups are autonomous and organize their own instructor courses.

**Conclusions:**

Training of autonomous Paediatric CPR groups with the collaboration and scientific assessment of an expert group is a good model program to develop Paediatric CPR training in low- and middle income countries. Participation of groups of different countries in the educational activities is an important method to establish a cooperation network.

## Background

The prognosis of cardiac arrest in children has improved in the last years, mainly due to the campaigns to extend the guidelines for prevention and cardiopulmonary resuscitation (CPR) [1, 2].

To perform a quality and early CPR is one of the most important prognostic factors of cardiac arrest [3]. Thus, the education in resuscitation of health professionals and general public in CPR is an important method to improve the results of CPR [4, 5].

Several articles have analyzed the characteristics of cardiac arrest in children [6, 7]. Cardiac arrest in children is a high mortality disease. Prognosis depends of several factors as the age, the previous state of health, the level of socio-economic development, the type and duration of cardiac arrest, and the quality of resuscitation [6, 7]. The mortality in children is higher in poorest populations [6].

Cardiac arrest in low-and middle-income countries (LMICs) has higher incidence and worse prognosis than in high-income countries, and these results are partly due to a low training level in cardiopulmonary resuscitation [6].

In many countries of Latin-America exist high differences in socio-economic level, children suffer malnutrition and health policies that develop health care for children are lacking. These factors increase the incidence of cardiopulmonary arrest in children and contribute to a bad prognosis [8].

The most useful methods of CPR training are structured courses that provide practice to small groups of students who perform manoeuvres on mannequins in simulated real cases [9–12]. There is an extensive experience with CPR courses worldwide, but most of them have been developed in high income countries [9–12].

The European Resuscitation Council (ERC), the American heart Association (AHA) and other CPR organizations have developed good educational programs, but the access to them in low-income countries is very scarce due to the high economic costs and the requirement to follow the educational model without changes neither adaptations [9, 11].

The education and training of the general population in prevention and basic CPR, and of health-care workers in advanced CPR has produced a significant reduction in mortality of cardiac arrest [4, 5, 9–12].

However, most of the education experiences performed in LMICs have been short-term projects depending on missions of foreign experts training local health professionals in resuscitation, and only few of them have achieved long-time maintenance mainly due to the lack of creation of an independent local educational program [13–17].

This was due mostly to the fact that these projects tried to exactly reproduce the European or American course without taking into consideration local country characteristics and they did not create an educational system to allow local health workers to continue the development of the project.

The Latin-American area has a huge children population with an even higher morbidity and mortality. Education in Pediatric CPR in most of Latin-American countries is not homogenous, and very few countries have a national coordination program [18].

The objective is to describe the methodology and results of a paediatric and neonatal CPR long-term training program in Latin-America. This program has been performed through an education and investigation international cooperation network that has the aim of creating long-term self-sufficient, independent and coordinated pediatric CPR educational groups adapted to local characteristics.

The educational model could serve for extending CPR to other developing countries.

## Methods

An investigation and education network (RIBEPCI) was developed to integrate clinical investigation and education on CPR in Latin-American countries and to create a long-term structure formed by autonomous but coordinated locals groups [19].

This network was funded by the Iberoamerican Science and Technology Program for Development (CYTED) with the scientific collaboration of the Spanish Paediatric and Neonatal Resuscitation Group (SPNRG). Financial support provided the teaching material necessary to launch the program and travel expenses of CPR instructors. The Spanish CPR group and instructors did not receive any fees. In addition, the SPNRG did not charge for certifying the quality of the course.

### General design and development of the program

An investigation and an educational project were designed to be developed simultaneously and independently, but groups from the education project were stimulated to participate also in the investigation project.

The main objective of the investigation project was to create a Latin-America pediatric cardiac arrest registry to identify the characteristics of cardiac arrest and the results of resuscitation in children in Latin-America.

This project enabled the performance of the first multicentric multinational study on cardiac arrest in children that included 543 cardiac arrest episodes in 124 hospitals from 16 countries [6]. The results of this study have been previously published [6, 20–24].

The main objective of the educational project was to create an autonomous stable long-term structure in each of the countries that participated with coordination and cooperation between them.

The educational project was designed and initiated by the Spanish group with the collaboration of the Honduran group [16]. In a previous article we published the first pilot experience in Honduras [16]. We used this pilot experience and adapted the educational model to develop the hole program.

It was developed in four countries consecutively using the same educational model. The educational team was initially formed by Spanish instructors. But the instructors trained in each country joined the educational team and participated in the development of the project in the next country, to reduce progressively the dependence of the Spanish group and to reach the cooperation and coordination between the groups included in the project.

Finally a Latin-America pediatric instructor course was developed with the participation of instructors from the six countries included in the program.

### Economical aspects

Mannequins and complementary teaching material were bought in Spain and sent to each country. The cost of material for each country was approximately 10.000 euros. The other cost funded by the program was the trip of the instructors. The local institution took care of the lodging and maintenance during the courses. The students of the first advanced life support course and instructor course did not pay anything for the courses, but they were asked to commit themselves to the future development of the program.

### Project phases in each country (Fig. 1)

An initial requirement to take part in the project was the participation of a local institution, which could be different depending on each country (university, state or private hospital, ministry of health and or education) that supported the project and undertook its maintenance.

**Fig. 1:**
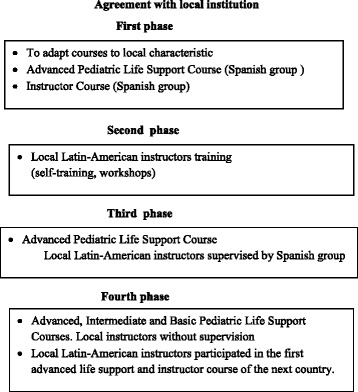
Phases of the project

The project in each country comprised four phases [16].

In the initial phase, the objective was to train a group of health professionals in advanced paediatric and neonatal CPR, and then as instructors in paediatric CPR to finally create a Pediatric CPR Group according to the local characteristics and needs. The first activity performed between the Spanish Coordination Group and the local group was to adapt the courses to local reality (for example causes of cardiac arrest, health organization, geographic and demographic characteristics, availability of material of resuscitation possibility to include the courses in university program, etc.).

Four to five CPR Spanish instructors travelled to each country to teach the courses (ALS and then Instructor course).

During the second phase, local instructors reinforced teaching skills by giving CPR workshops to residents and medical students. CPR workshops were part of the whole advanced CPR course (e.g. instrumental airway management and oxygen bag and mask ventilation during paediatric CPR) [16].

In the third phase, the local instructors gave a advanced paediatric and neonatal CPR course supervised by the Spanish group [16].

In the final phase, the local group programmed, delivered, and certified CPR courses independently. The Spanish group only provided scientific support but no specific accreditation.

### Courses

Several courses were designed: Paediatric Advanced Life Support (ALS) course, Pediatric Basic Life Support (BLS) course, Paediatric Intermediate Life Support (ILS) course and Pediatric Advanced Life Support Instructor course.

The ALS course of 20–24 h of duration was an on-site course combining theory and practice and using a teaching approach based on advanced interactive simulation with mannequins [10, 16]. Theoretical classes were interactive lectures. All practical classes included individual supervised practice of each student Skill stations to learn the techniques in instrumental airway management, vascular access, arrhythmias, and trauma management). The maximum number of students in each practical group was six. Interactive case simulation scenarios where CPR was performed in a real time fashion (basic CPR, instrumental airway management, vascular access, arrhythmias, initial management of trauma patients, neonatal CPR and integrated advanced resuscitation) (Table 1). The schedule of an ALS was showed in Table 2. When case management finished, self-evaluation and an evaluation between students and instructor was performed. Local case etiology, environmental factors and number of rescuers were taken into account when designing simulation.

**Table 1:** Advanced life support program course

I. Theoretical classes	1. Cardiopulmonary arrest. Concepts and prevention in children. 2. Paediatric basic life support. 3. Instrumental airway management and ventilation. 4. Vascular access, drugs and fluids. 5. Monitoring, diagnose and treatment of arrhythmias. 6. Neonatal resuscitation. 7. Post-resuscitation stabilization: initial management and stabilization of paediatric trauma patients; ethics. 8. Integrated paediatric advanced resuscitation.
II. Practical classes	1. Basic life support (infant and child). 2. Instrumental airway management and ventilation: Guedel cannula, suction, bag and mask ventilation, endotracheal intubation. Hands-on practice and case simulation. 3. Vascular access, drugs and fluids. Intravenous and intraosseus needle insertion in arm, leg and chicken’s bone. Drug doses calculation, preparation and administration. Intratracheal tube drug administration. Hands-on practice. 4. Monitoring, diagnose and treatment of arrhythmias. Interpretation of EKG, diagnosis of arrhythmias and defibrillation technique. Hands-on practice and case simulation. 5. Initial management and stabilization of paediatric trauma patients. Helmet withdrawal, collar and spinal table positioning. Hands-on practice and case simulation. 6. Neonatal resuscitation. Umbilical intravenous access. Leadership and team coordination. Hands-on practice and case simulation. 7. Integrated advanced resuscitation. Leadership and team coordination. Hans-on practice. Case simulation.

**Table 2:** Schedule of advanced life support program course

Title	Educational activities	Duration Minutes
First session		
Introduction. Preliminary test	Evaluation	45
Cardiopulmonary arrest. Concepts and prevention in children.	Lecture	30
Paediatric basic life support.	Lecture	45
Pediatric basic life support with infant and children manikins	Practical	180
Second session		
Instrumental airway management and ventilation.	Lecture	45
Instrumental airway management and ventilation	Practical	150
Vascular access, drugs and fluids.	Lecture	45
Vascular access, drugs and fluids.	Practical	90
Third session		
Monitoring, diagnose and treatment of arrhythmias.	Lecture	45
Monitoring, diagnose and treatment of arrhythmias.	Practical	90
Post-resuscitation stabilization	Lecture	30
Ethics and resuscitation	Lecture	30
Four session		
Neonatal resuscitation.	Lecture	45
Neonatal resuscitation.	Practical	120
Trauma and cardiac arrest	Lecture	45
Trauma and cardiac arrest	Practical	90
Fifth sessions		
Resuscitation algorithms Team work	Lecture	45
Integrated advanced resuscitation Practical evaluation	Practical	180
Final evaluation Evaluation of the course	Evaluation	45

BLS course of 4–6 h was developed by the local group using the first part of the advanced CPR course, adapted to the characteristics of the students. Automatic defibrillation (AED) was not included in this course because none of the countries had public access to AED at that moment.

An intermediate Life Support Pediatric course of 10–14 h of duration was designed adapted to the needs of health care professionals of the countries. This course included basic CPR, airway management without intubation, peripheral and intraoseous access, drug treatment and team work and coordination of the resuscitation. Depending of the characteristics of the students the Intermediate CPR course also included initial neonatal resuscitation, trauma management and diagnosis and treatment of arrhythmias.

The ALS instructor course was designed to train instructors; the participants were doctors who had previously been certified in Advanced Paediatric and Neonatal CPR. The teaching approach involved advanced interactive simulation with theoretical classes complemented by practical ones using mannequins. Using the same methodology as in the CPR course, instructor students were asked to design scenarios and run simulation cases. Both self-evaluation and instructor evaluation and feedback were incorporated [25] (Table 3).

**Table 3:** Advanced life support instructors program course

I. Theoretical classes	1. Types and organization of Paediatric life support courses. 2. Teaching techniques and educational methods. 3. Mock codes preparation. 4. Evaluation methodology. 5. Practice demonstration.
II. Practical classes	1. Techniques of oral expression: public speaking 2. Basic life support. 3. Instrumental airway management and ventilation. 4. Vascular access, drugs and fluids. 5. Monitoring, diagnose and treatment of arrhythmias. 6. Neonatal resuscitation. 7. Trauma an resuscitation 8. Integrated advanced resuscitation.

Characteristics of the classes and material have been previously published [10, 16, 25].

## Results

### Initial results

The educational project was sequentially developed in four countries (Honduras, Guatemala, Dominican Republic and Mexico). Two more groups participated: the Spanish group as coordinating group and a Panamanian group, which had previously developed its local educational program, but joined in to collaborate with the educational activities in the other countries.

Five Pediatric Resuscitation Groups were created (two in Honduras and one in Guatemala, Dominican Republic and Mexico).

Six Pediatric CPR instructor courses were developed. The last one was an international Latin-American instructor course. Ninety-four students were accredited as Pediatric CPR instructors at the end of 2015 (Table 4).

**Table 4:** Instructors courses

	Advanced Life Support Instructor courses	
	Courses	Students
Honduras	2	32
Guatemala	1	12
Dominican Republic	2	32
Mexico	1	18
Total	6	94

From the beginning of the project 2009 to the end of 2015 a total of 182 Pediatric Basic, Intermediate and Advanced Life Support courses were performed and 3637 students were certified.

The Table 5 summarises the type of courses and student accredited by country. Sixty-four Paediatric BLS Courses (1409 students), 29 Paediatrics ILS courses (626 students) and 89 Paediatric ALS courses (1804 students) were performed.

**Table 5:** Basic, intermediate and advanced paediatric life support courses

	ALS courses		ILS courses		BLS courses	
	Courses	Students	Courses	Students	Courses	Students
Honduras	32	612	3	46	3	44
Guatemala	38	750	4	91	38	848
Dominican Republic	8	195	7	137	7	102
México	11	247	15	352	16	415
Total	89	1804	29	626	64	1409

*ALS* advanced life support, *ILS* intermediate life support, *BLS* basic life support

Basic Pediatric CPR courses were imparted to general population, schools (children and teachers), firemen and policemen.

Intermediate Pediatric CPR courses were imparted to health professionals (nurses, general physicians, primary health care and community hospital pediatricians and Medicine and Nursing students).

Advanced Pediatric CPR course were given to Pediatrics residents and pediatric and nurse staff of Emergency and Pediatric Critical Care Units.

Independent Pediatric CPR associations were created in Honduras, Guatemala and Dominican Republic as a non-profit scientific groups with the scientific collaboration and assessment of the GERCPN. In Mexico an alliance was established with the Centro Nacional para la Salud de la Infancia y la Adolescencia (CeNSIA) that works with community hospitals in rural regions of Mexico.

## Discussion

In 2013 a consensus conference recommended the creation of a research agenda for adult cardiac arrest and resuscitation. One of the conclusions of that conference was that in LMICs there is a need to understand the epidemiology, infrastructure and systems context, level of training, and cost effective care to improve the results of resuscitation [26, 27]. However in that conference the specific characteristics of pediatric resuscitation were not analyzed. A global pediatric conference should be developed to create a specific pediatric agenda and to study which are the most cost-effective strategies to improve outcomes in LMICs.

We describe the first educational program in Pediatric Cardiopulmonary Resuscitation designed and developed to reach a long-time independent maintenance of resuscitation training in low- and middle-income countries through the creation of local autonomous educational structures [28]. Our experience in Latin-America may serve for other countries, adapting the model to the specific charcateristics. Besides, the educational and investigational methodology can be used in projects on other health areas.

The most important characteristics of our educational program are:

### Organization model

Our model involved the scientific collaboration of a Spanish Resuscitation group that has a long experience in CPR training with local healthcare professionals of each country. We organized local educational groups in each country and these groups were the responsible to maintain autonomously the educational program for the long run.

For this reason, an initial requirement was the participation of a local institution that could support the maintenance of the project.

### Educational methodology

In the first pediatric resuscitation and instructor courses the instructors of the coordinating group offered a model to follow, created a style, and resolved many of the doubts that arise when giving a course.

To avoid the continued dependence of the coordinating group it was essential to train a sufficient pool of instructors in each country and train them sufficiently so that they could in turn educate new instructors and extend the training chain.

Supervision of the new instructors was essential to ensure the quality of training, thus requiring the instructors of the coordinating group to travel to monitor the first courses taught by each educational group.

### Adaptation to local characteristics

Unlike other educational programs ours did not impose a specific recommendation of resuscitation. Each country could decide to follow the recommendations it considered most appropriate for its characteristics.

We think it is very important that each educational group modifies and adapts the resuscitation courses to the local conditions, because they are who know the needs and characteristics of the population. The differences between courses depend more on the educational needs of the students who received the course (for example health personnel of rural hospitals or residents of Pediatrics) than on the country.

Other groups have recently adopted this strategy to contextualize the Pediatric Emergency Assessment Recognition and Stabilization courses of the American Heart Association to the features of the LMICs [29].

### Economical aspects

The economic is one of the most important issues in a CPR educational program. Some authors have remarked that health care systems with limited resources should include CPR training only after considering the economic and ethical implications [30].

One problem of courses accredited by AHA or ERC is that these organizations demand very high accreditation costs. The prices of accreditation, copyright of resuscitation courses and teaching materials, and are too expensive for many countries with low economic level. Moreover they demand a strict adherence to its CPR recommendations and course programs, regardless of the characteristics of each country. For these reasons in developing countries very few health professionals can access to a high-quality training program.

We got an initial public funding to start the project. This is necessary because the costs of mannequins are high. But our experience showed that with a not very important financial support it is possible to create an educational program that integrates teaching and research in LMICs. We advocate for the elimination of the payment for accreditation for countries with low economic resources. This fact could allow them to develop and maintain their training programs in resuscitation.

A very important feature of our system is that training was non-profitable, and that its main objective was to extend the Pediatric CPR training to all health and non-health levels. Unfortunately, in some countries with limited resources there are some CPR training groups but they are exclusively dedicated to give courses to the very small number of students who can afford the courses (as these have a high price), and try to prevent the creation of other educational groups that can compete with them.

A key objective of our educational program is that each local group is autonomous and self-financing. Each group in each country must find the most convenient sources of funding and decide the best way to compensate instructors to ensure the maintenance and extension of the program, including the acquisition and replacement of teaching materials. We think that resuscitation instructors in developing countries should receive fees for their work but the cost would be low in this non-profit context. However each country should analyze its possibilities to allow the self-sufficiency and sustainability of the educational program.

### Multinational collaboration

A differentiating aspect of our program is that it has developed a network of educational collaboration in pediatric CPR so that instructors of each country are involved in the formation of the next group creating an increasingly broad multinational cooperation group in which they share very different experiences. This allowed that the dependence of the coordinating group was diminishing until finally it acted only as a general coordinating bond between the groups.

The next objectives of the RIBEPCI Network are first to achieve a stable funding to extend the program to other countries, and to create a stable network of cooperation in teaching and research in cardiac arrest and resuscitation in Latin America that encourages multinational cooperation to improve results in health.

### Limitations

Our program was developed in several nearby countries of Central America and this fact allowed the communication between the members. Each program must analyze regions features to exploit the possibilities of cooperation between different countries.

## Conclusions

To create autonomous Paediatric CPR groups with the collaboration of an expert group, is a good model to develop Paediatric CPR training in low- and middle income countries. Participation of groups of different countries in the educational activities is an important method to establish a cooperation network. International cooperation and coordination is essential to develop long-term educational CPR training throughout the world.

## Abbreviations

AED: Automatic defibrillation
ALS: Advanced life support
BLS: Basic life support
CPR: Cardiopulmonary resuscitation
ILS: Intermediate life support
LMICs: Low-and middle-income countries
